# Size Matters: Assessing Optimum Soil Sample Size for Fungal and Bacterial Community Structure Analyses Using High Throughput Sequencing of rRNA Gene Amplicons

**DOI:** 10.3389/fmicb.2016.00824

**Published:** 2016-06-02

**Authors:** C. Ryan Penton, Vadakattu V. S. R. Gupta, Julian Yu, James M. Tiedje

**Affiliations:** ^1^Faculty of Science and Mathematics, College of Integrative Sciences and Arts, Arizona State UniversityMesa, AZ, USA; ^2^Arizona State University Applied and Functional Microbiomics Institute, Arizona State UniversityMesa, AZ, USA; ^3^CSIRO AgricultureGlen Osmond, SA, Australia; ^4^Center for Microbial Ecology, Michigan State UniversityEast Lansing, MI, USA

**Keywords:** DNA extraction, fungal community, microbial ecology, sample size, microbial diversity

## Abstract

We examined the effect of different soil sample sizes obtained from an agricultural field, under a single cropping system uniform in soil properties and aboveground crop responses, on bacterial and fungal community structure and microbial diversity indices. DNA extracted from soil sample sizes of 0.25, 1, 5, and 10 g using MoBIO kits and from 10 and 100 g sizes using a bead-beating method (SARDI) were used as templates for high-throughput sequencing of 16S and 28S rRNA gene amplicons for bacteria and fungi, respectively, on the Illumina MiSeq and Roche 454 platforms. Sample size significantly affected overall bacterial and fungal community structure, replicate dispersion and the number of operational taxonomic units (OTUs) retrieved. Richness, evenness and diversity were also significantly affected. The largest diversity estimates were always associated with the 10 g MoBIO extractions with a corresponding reduction in replicate dispersion. For the fungal data, smaller MoBIO extractions identified more unclassified *Eukaryota incertae sedis* and unclassified glomeromycota while the SARDI method retrieved more abundant OTUs containing unclassified Pleosporales and the fungal genera *Alternaria* and *Cercophora*. Overall, these findings indicate that a 10 g soil DNA extraction is most suitable for both soil bacterial and fungal communities for retrieving optimal diversity while still capturing rarer taxa in concert with decreasing replicate variation.

## Introduction

The complex structural and spatial physico-chemical heterogeneity of soils likely influences microbial community structure, particularly over varying spatial scales. Microbial populations can be preferentially localized in microhabitats, e.g., rhizosphere, detritusphere, drillosphere, aggregatosphere, pores, organic matter coatings, etc. that provide suitable habitat requirements (Hattori, 1988; Bailey et al., 2013; Vos et al., 2013). Spatial heterogeneity due to carbon and nutrient availability and redox potential gradients also promote diversity by providing specific niches and creating ecological opportunities (Rainey and Travisano, 1998; Gupta and Germida, 2015). Given this complexity, soil microbial biology was often treated as a “black box” (Tiedje et al., 1999) but, with the advent of molecular methods, that box began to open to better reveal the players and their activities as influenced by management and environmental attributes.

Sample size, along with replication, sampling design, DNA extraction, and molecular analyses (currently amplicon sequencing methods), affect measures of community structure including alpha and beta diversity, dispersion, and hence comparisons among treatments and among experiments. Given the spatial heterogeneity of soil, sample size should be large enough to encompass all the significant microhabitats of the ecological unit under study so that larger drivers of biological structure can be discerned.

While sampling strategies for high-throughput amplicon sequencing studies often focus on the number of replicates taken in order to increase the power of statistical analyses, they often ignore the size of the soil sample used for DNA extraction. Prior studies have given some general insight into the influence of sample size on community structure results. Ellinsøe and Johnsen (2002), using denaturing gradient gel electrophoresis (DGGE), found larger variations in community structure among replicates with small sample sizes (0.01 and 0.1 g) versus larger samples (1.0 and 10 g). They concluded that small soil samples harbor bacterial communities that are missed in larger soil samples. Ranjard et al. (2003), using automated ribosomal intergenic spacer analysis (ARISA) on soil sample sizes from 0.125 to 4 g, found that bacterial community structures were similar for all sample sizes, but fungal communities had higher replicate variation, particularly in small sample sizes. They suggested that small soil samples more accurately reflect the fungal composition than that observed in larger soil samples. The central conclusion drawn from this study was that large soil samples are most suitable for the description of the overall soil community but large numbers of small samples are more appropriate for a determination of local microbial diversity.

Archaeal community structure assessed by DGGE was more similar among 10 g replicates than 0.1 and 1 g extraction sizes (Nicol et al., 2003). Their general conclusion agreed with Ranjard et al. (2003) in that rare members of the archaeal community would likely not be observed using large samples and that an extensive microsampling approach was necessary to assess the rare components that are present only in microenvironments. Sample size also affects ecofunctional gene analysis; Stres et al. (2004) found increasing convergence of RFLP profiles of *nosZ* (nitrous oxide reductase) as sample sizes reached 1 to 3 g samples, which then allowed distinctions among sites. Kang and Mills (2006), using DGGE and soil extraction sizes ranging from 0.01 to 10 g from a native temperate tallgrass meadow, found that replicate dispersion was lowest for the bacterial community in the 0.25 to 10 g samples while the fungal communities were clustered in the 0.1 to 0.25 g samples. They concluded that 0.25 g was optimal for the assessment of both bacterial and fungal communities from a single DNA extraction. These studies show that small samples detect more rare members but resulted in more variation among replication and hence less ability to distinguish among treatments or conditions. No studies have evaluated effects of sample sizes, ranging from 0.25 g to 100 g, using the much higher through resolution and sampling depth provided by high throughput sequencing. However, Song et al. (2015) did find that with increasing sample size there was an increase (although non-significant) in the average OTU richness from fungal amplicon (ITS) sequence data for prairie and forest soils in USA in soil sample sizes ranging from 0.25 g to 10 g.

The aim of this study was to evaluate the effect of microscale heterogeneity by utilizing a range of soil sample sizes – 0.25 to 100 g – for DNA extraction on both bacterial and fungal community structure measures as determined by high-throughput amplicon sequencing of the bacterial 16S and the fungal 28S rRNA genes. An optimum sample size was found for overall community structure, replicate dispersion, within replicate similarity and variations in diversity indices among soil extraction sizes and methodologies. Pairwise comparisons of extractions were used to determine the presence of large discrepancies in the relative abundances of specific taxa due to sample size.

## Materials and Methods

### Sample Description and DNA Extraction

Four GPS locations in one agricultural field located at Avon in South Australia (S34 13.981, E138 18.586) were sampled during the non-crop season in March, 2012. At each location a 15 m × 12 m area was marked as a field replicate (see Supplementary Figure S1 for sampling design). The soil type is Luvic Calcisol and sandy to sandy loam in texture (Lithocalcic Calcarosol) (Northcote et al., 1975). Soil physio-chemical properties were: clay 17%, sand 51%, silt 32%, organic C 1.6%, total N 0.15%, and pH (water) 8.3. The site was cropped in cereals (wheat, barley, or oats) for at least 5 years. Two independent collections of three 40 mm diameter cores, at randomly selected points (∼490 g soil each), were taken at each of the four GPS locations within the field (Supplementary Figure S1). In order to reduce large-scale heterogeneity while preserving aggregate structure and retaining microscale heterogeneity, each group of three cores was gently mixed yielding a composited sample representing each of the four field replicate locations. Large un-decomposed plant material and stones were removed. The sandy loam texture of the soil does not allow formation of large aggregates or clods that requires sieving of soil. Sub-samples of different sizes (0.25 g, 0.5 g, 1.0 g, 10 g, and 100 g) were taken from each of the two composited samples consisting of three cores, for a total of four samples for each of the four field locations, resulting in 16 samples per sample size for MoBIO extraction (Supplementary Figure S1). For SARDI DNA extraction, one sample from each composite was taken, resulting in 8 samples for SARDI 10 g and 100 g extractions. Soil samples were immediately placed on ice in a cooler, transported to the laboratory and stored at -20°C until lyophilized for SARDI extraction, or until shipped on dry ice to Michigan State University for DNA extraction as follows. Genomic DNA was extracted from 0.25 g soil sub-samples using the MoBIO PowerSoil DNA Isolation Kit. 1.0 g, 5 g, and 10 g soil samples were extracted using the MoBIO PowerMax Soil DNA Isolation Kit following manufacturer’s instructions. The additional 10 g and 100 g soil samples remaining in Australia were extracted by the South Australian Research and Development Institute (SARDI, Adelaide, AU, USA) Root Disease Testing Service (Ophel-Keller et al., 2008). SARDI utilizes a bead-beating method and has been demonstrated to be an effective method for quantifying plant roots (Haling et al., 2011; Huang et al., 2013) and soil fungi (Simpson et al., 2011; Bithell et al., 2013). In total there were 16–0.25 g, 16–1.0 g, 16–5 g, 8–10 g (MoBIO), 8–10 g (SARDI), and 8–100 g (SARDI) soil DNA extractions used as templates for PCR amplification at the Center for Microbial Ecology at Michigan State University.

### 28S and 16S rRNA Gene Amplification

Fungal 28S rRNA gene amplicons were generated using primers LR3/LR0R^1^ (Liu et al., 2012) according to previously published protocols (Penton et al., 2013, 2014). Quadruplicate amplification replicates were pooled and gel purified using the Qiagen Gel Purification Kit following band excision then further purified using the Qiagen PCR Purification Kit. Following adapter ligation, amplicons were sequenced by the Utah State University CIB Genomics Core Lab on the 454 Titanium platform.

Bacterial 16S rRNA genes were amplified using the dual index paired-end approach for the Illumina MiSeq platform (Kozich et al., 2013). Briefly, each primer consisted of an Illumina adapter, an 8-nt index sequence, 10-nt pad sequences, a 2-nt linker and the 16S V4 primer sequence forward (CCTACGGGAGGCAGCAG) or reverse (GGACTACHVGGGTWTCTAAT). Amplification was performed on a 96-well plate using AccuPrime Pfx SuperMix reagents and library clean-up and normalization was performed using the Invitrogen SequalPrep Plate Normalization Kit. The library QC was performed using a KAPA Biosystems qPCR kit and by obtaining a bioanalyzer trace using the Agilent Technologies HS DNA kit. Sequencing was done at Michigan State University’s Research and Technology Support Facility.

### Sequence Processing and Statistics

Raw 28S rRNA gene sequences were processed for minimum length (400 bp), quality (*Q* > 20), primer match and barcode sorting using the RDP pyrosequencing pipeline. Chimeras were identified and removed using UCHIME (Edgar et al., 2011) in *de-novo* mode and the remaining sequences were randomly re-sampled to 4,300 sequences per sample using MOTHUR (Schloss et al., 2009). Three samples were discarded that did not meet the minimum resampling depth. The remaining 266,600 sequences were aligned then clustered at 5% nucleotide dissimilarity and representative sequences generated for each OTU using RDP tools hosted on the Michigan State University High Performance Computing Center servers^2^. The RDP Fungal Classifier^3^ based on training set 11 was used for classification of each cluster representative sequence.

Bacterial 16S rRNA gene amplicons were sequenced on the Illumina MiSeq platform (2 bp × 250 bp paired end reads). Raw reads were assembled using a modified PandaSeq (Cole et al., 2014) with a minimum overlap of 50 bp, minimum and maximum lengths of 220 and 280, respectively, and a minimum Q score of 28 as determined by defined community analysis using RDP tools (Fish et al., 2013). All computation was performed on the MSU High Performance Computing Center servers. UCHIME (Edgar et al., 2011) was used to identify and remove chimeras followed by resampling at 23,000 sequences per sample using MOTHUR (Schloss et al., 2009), alignment then clustering at 3% nucleotide dissimilarity. Representative sequences were classified using the RDP Classifier with training set 9 at 80% confidence.

Raw cluster abundances were Hellinger transformed and a Bray-Curtis dissimilarity matrix (+1) was constructed, statistical analyses performed and diversity estimates calculated using PRIMER-E (Clarke and Gorley, 2006). Statistical analyses were based on four replicates from each of the four field GPS locations (*n* = 16), except for SARDI 10 g and 100 g extractions (*n* = 8). Cluster analysis was performed with the Similarity Profile analysis (SIMPROF) test (Clarke et al., 2008). Significant differences in community structure were tested using Permutational Multivariate Analysis of Variance (PERMANOVA) (Anderson, 2001) and Analysis of Similarity (ANOSIM) (Clarke, 1993). Sample replicate dispersion was tested by Permutational Analysis of Multivariate Dispersions (PERMDISP) (Anderson et al., 2006) and a test for Multivariate Dispersion (MVDISP). ANOVA statistics for Shannon diversity (H’), Pielou’s Evenness (J), Margalef’s Richness (d) and the number of individuals (N) were performed using Minitab 16 (Minitab Inc, USA). Sequences were deposited in the European Nucleotide Archive^4^ under study PRJEB8081 with accession numbers ERS632772–632841 and ERS671660–ERS671724.

## Results

### Sequencing

A total of 591,120 fungal 28S rRNA gene sequences were retrieved after initial processing for quality, length, and matches to the forward primer sequence; 2.8% of all sequences were identified as chimeras and removed prior to re-sampling. Clustering at 5% nucleotide dissimilarity on 4,300 sequences per sample yielded 23,431 clusters of which 14,352 were singletons or doubletons. For the bacterial 16S rRNA genes, a total of 5,501,355 raw paired end reads produced from 70 samples yielded 4,130,058 assembled and quality-filtered reads. A total of 1.3% of filtered reads were identified as chimeras and removed. Clustering at 3% nucleotide dissimilarity on 23,000 sequences per sample yielded 18,355 OTUs of which 4736 were singletons and 1840 were doubletons.

### Community Differences with Sample Size and Extraction Method

For both the bacterial and fungal communities, Margalef’s richness (d, ANOVA, 28S: *F* = 11.25, *P* < 0.001, 16S: *F* = 18.42, *P* < 0.001), Pielou’s Evenness (J’, 28S: *F* = 6.3, *P* < 0.001, 16S: *F* = 10.81, *P* < 0.001), Shannon Diversity (H’, 28S: *F* = 9.1, *P* < 0.001, 16S: *F* = 15.65, *P* < 0.001) and the number of individuals (N, 28S: *F* = 7.9, *P* < 0.001, 16S: *F* = 15.58, *P* < 0.001) were significantly different among extraction sizes in both datasets, with the highest values always associated with the 10 g MoBIO extraction (**Table 1**).

**Table 1:** Diversity indices for 28S and 16S rRNA genes according to sample extraction size for Margalef’s richness (d), Pielou’s evenness (J’), Shannon Diversity (H’), and the overall number of individuals (N) with ANOVA grouping with Tukey’s test at 95% confidence shown by superscript letters.

	28S				16S			
Size	*d*	*J’*	*H’*	*N*	*d*	*J’*	*H’*	*N*
0.25 g	239.6^B^	0.981^C^	7.09^B^	313^C^	648.3^B^	0.976^B^	8.10^B^	506.3^BC^
1 g	241.1^B^	0.983^BC^	7.12^B^	322^BC^	544.2^C^	0.977^B^	7.92^C^	469.1^C^
5 g	269.3^A^	0.985^AB^	7.25^A^	345^AB^	660.0^B^	0.977^B^	8.13^B^	514.4^B^
10 g	**285.5**^A^	**0.987**^A^	**7.33**^A^	**365**^A^	**779.6**^A^	**0.979**^A^	**8.33**^A^	**572.7**^A^
10 g (SARDI)	238.2^B^	0.982^BC^	7.10^B^	317^BC^	766.3^A^	**0.979**^A^	8.31^A^	566.3^A^
100 g (SARDI)	237.1^B^	0.982^BC^	7.09^B^	317^BC^	715.5^AB^	0.977^AB^	8.22^AB^	537.8^AB^

*The highest values in each column are bolded for reference*.

Significant differences in fungal and bacterial community composition were identified from PERMANOVA analysis among soil sample sizes (28S: *F* = 2.18, *P* = 0.001, 16S: *F* = 2.79, *P* = 0.001) and among replicates (28S: *F* = 1.41, *P* = 0.001, 16S: *F* = 1.27, *P* = 0.013) but not with the interaction terms of size × replicate (28S: *F* = 0.86, *P* > 0.10, 16S: *F* = 0.94, *P* > 0.10). Permutational dispersion (PERMDISP) revealed significant overall differences in sample dispersion among extraction sizes (28S: *F* = 43.6, *P* = 0.001, 16S: *F* = 14.47, *P* = 0.001) with dispersion values decreasing with increasing sample extraction size (28S: ANOVA, *F* = 43.60, *P* = 0.001, 16S: *F* = 14.5, *P* < 0.001) (**Table 2**). Decreasing multivariate dispersion indices (MVDISP) with increasing extraction size for both 28S and 16S datasets was also found. PERMANOVA-based similarities of within replicate groups for 16S data also increased with sample size from 34.5% in 0.25 g to 48.8% in 100 g (**Table 2**). For the fungal data the within-group similarities increased from 62.5% in 0.25 g to 67.7% in 100 g, although there was a decrease associated with the 1 and 5 g samples. Dispersion among the sub-replicates was calculated using PERMDISP and the four values for each extraction size were averaged (Dmean Rep, **Table 2**). These dispersions showed that similarity among sub-samples increased as extraction size increased. The number of total OTUs retrieved was significantly different among sample sizes (ANOVA, 28S: *F* = 10.91, *P* < 0.001, 16S: *F* = 17.63, *P* < 0.001) as were the non-singleton/doubleton OTUs (ANOVA, 28S: *F* = 5.08, *P* = 0.001, 16S: *F* = 14.47, *P* < 0.001).

**Table 2:** 28S and 16S rRNA gene results from the permutational dispersion (PERMDISP) test showing dispersion means (Dmean) and standard errors (SE) for the extraction size groups.

	28S					16S				
Extraction Size	Dmean	SE	SIM	MVD	Dmean Rep	Dmean	SE	SIM	MVD	Dmean Rep
0.25g	44.66^A^	0.70	34.5%	1.21	**38.9**	25.62^BC^	0.48	62.5%	1.03	22.3
1g	44.40^A^	0.91	34.4%	1.24	36.3	**29.12**^A^	1.55	**55.4%**	**1.69**	23.5
5g	**44.68**^A^	0.36	**34.3%**	**1.27**	37.4	27.70^AB^	0.86	59.4%	1.34	**23.9**
10g	37.79^B^	0.45	37.3%	0.34	28.4	23.44^CD^	0.27	64.6%	0.72	17.7
10g (S)	38.43^B^	0.59	42.3%	0.36	29.5	22.25^D^	0.35	66.4%	0.36	16.7
100g (S)	**33.54**^C^	0.26	**48.8%**	**0.07**	**25.6**	**21.40**^D^	0.39	**67.6%**	**0.19**	**15.9**

*SARDI extractions are indicated by (S). Dmean Rep data are the mean dispersions from the four sub-plots according to extraction size. Superscript letters indicate ANOVA grouping with Tukey’s test at 95% confidence. Columns without grouping superscripts are not testable via ANOVA. SIM = within group average similarity determined by PERMANOVA. MVD = Multivariate dispersion index. Bolded values indicate highest and lowest values for each column. Columns lacking ANOVA grouping were not testable*.

**FIGURE 1:**
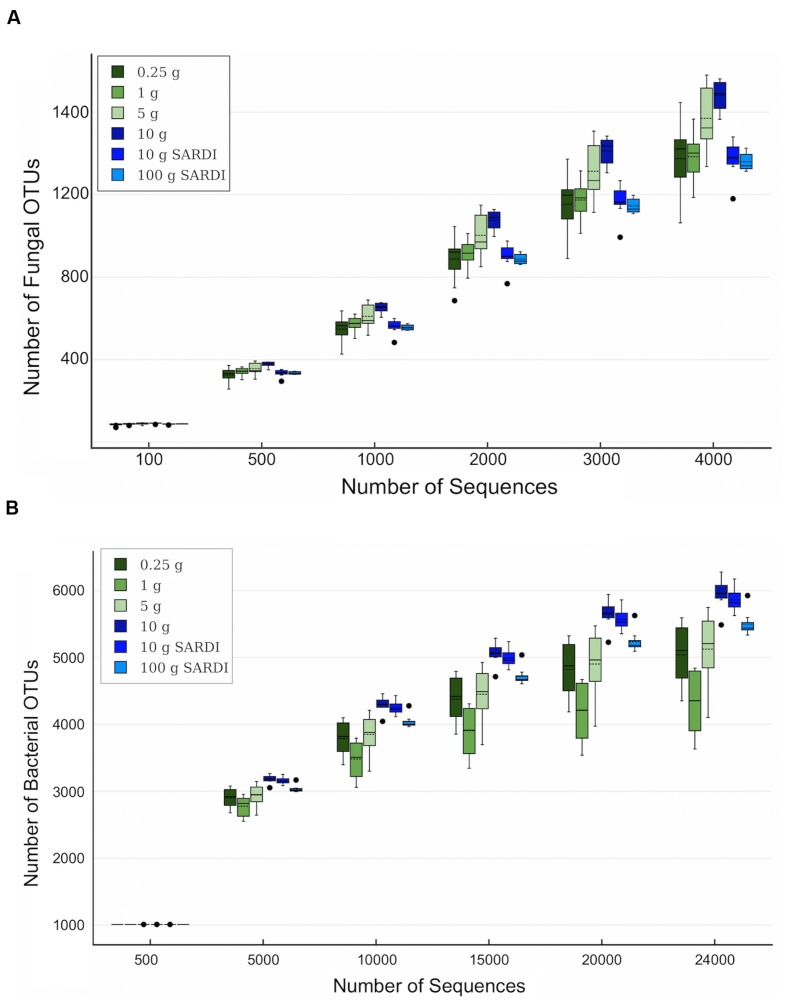
**Rarefaction data box plots based on (A) fungal and (B) bacterial rRNA OTU data at 5 and 3% sequence dissimilarity, respectively**. Solid line is the mean, dashed line is the median, dots indicate outliers among replicates.

For both 28S and 16S the largest number of OTUs was associated with the 10 g SARDI and 10 g MoBIO extractions, respectively (**Table 3**). After the removal of singleton-doubleton OTUs, the bacterial data again showed that 10 g MoBIO resulted in the highest number of OTUs (total and unique sequences), though by a small margin over 10 g SARDI. For the fungal data, the highest number of non-singleton-doubleton OTUs originated from the 1 g sample, with the 10 g MoBIO a close second. Fungal OTU-based rarefaction data (**Figure 1A**) showed smaller replicate variance in the 10 g, 10 g SARDI and 100 g SARDI sequence data. The 10 g extractions consistently showed higher coverage, especially compared to the 0.25 g and 1 g samples. Bacterial OTU-based rarefaction data (**Figure 1B**) illustrated the same trend with the additional observation that the 0.25 g, 1 g, and 5 g extractions especially showed a trend toward earlier saturation.

**Table 3:** Average number of total rRNA OTUs and of the non-singleton or doubleton (Non-S/D) OTUs retrieved from each sample size.

	Total OTUs		Non-S/D OTUs	
Extraction Size	28S	16S	28S	16S


0.25g	1379^A^	3960^A^	**319**^A^	**1722**^A^


1g	1394^A^	**3314**^B^	**366**^B^	1769^AB^


5g	1576^B^	4016^A^	339^ABC^	1764^A^


10g	1394^B^	**4701**^C^	360^BC^	**1869**^B^


10g (SARDI)	**1685**^A^	4636^C^	330^AC^	1868^B^


100g (SARDI)	**1366**^A^	4358^AC^	334^ABC^	1785^AB^

*ANOVA grouping with Tukey’s test at 95% confidence are denoted by superscript letters. The largest and smallest values in each column are bolded for reference*.

**FIGURE 2:**
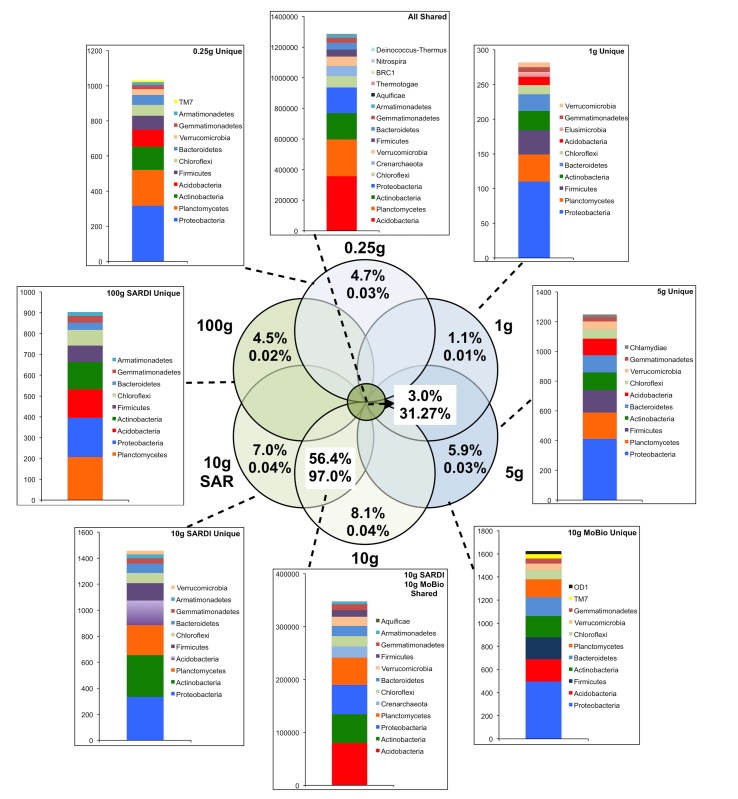
**Sequence abundances of the top most abundant unique OTUs for each sample size and their corresponding classification for the bacterial 16S rRNA gene sequences**. OTUs that were shared among all sample sizes are shown in the “All Shared” inset with those shared between the SARDI 10 g and MoBIO 10 g in an additional inset. Within the Venn diagram, top numbers indicate the percent of total OTUs while the bottom represents the percent of total sequences.

**FIGURE 3:**
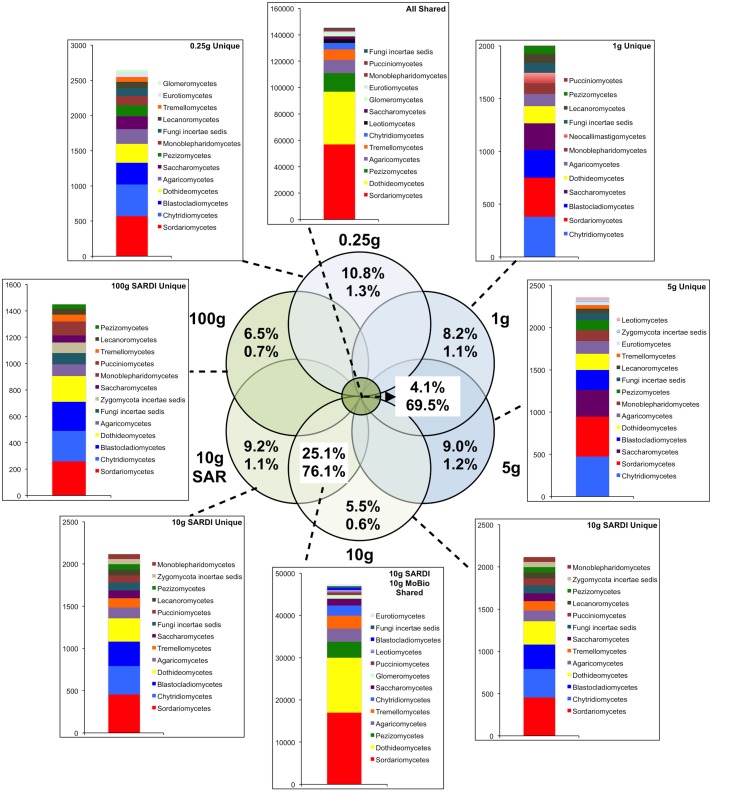
**Sequence abundances of the top most abundant unique OTUs for each sample size and their corresponding classification for the fungal 28S gene sequences**. OTUs that were shared among all sample sizes are shown in the “All Shared” inset with those shared between the SARDI 10 g and MoBIO 10 g in an additional inset. Within the Venn diagram, top numbers indicate the percent of total OTUs while the bottom represents the percent of total sequences.

In total, 31.3% of the bacterial (**Figure 2**) and 69.5% of the fungal (**Figure 3**) sequences were shared among all extraction sizes, including both extraction methods. For bacteria, the most abundant unique sequences for any one extraction generally belonged to the Proteobacteria, Planctomycetes, Acidobacteria, Actinobacteria, and Firmicutes, though they comprised less than 0.05% of all sequences. For the fungi, unique sequences were somewhat less rare, though they did not exceed 1.3% of the total. The most abundant unique fungal sequences belonged to the Chytridiomycetes, Sordariomycetes, Dothideomycetes, and Blastocladiomycetes. Among these, the Dothideomycetes appear to be more frequently common than unique in any one size-extraction method sample. In addition, the proportion of unique OTUs to unique sequences in any one sample indicates that these unique sequences were relegated to low abundance OTUs. The 0.25 g and 10 g MoBio samples shared 98.0% of bacterial and 83.8% of fungal sequences contained within 60.0 and 30.5% of the total OTUs, respectively.

**FIGURE 4:**
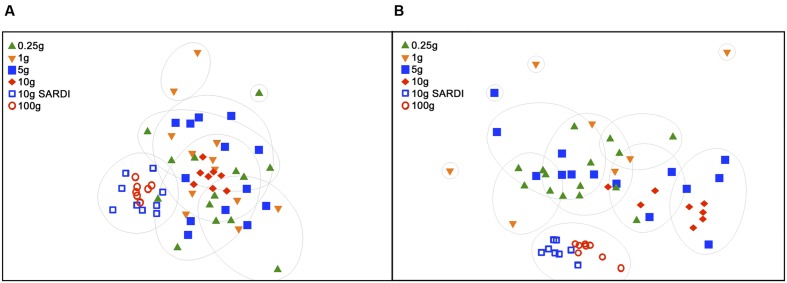
**Non-metric dimensional scaling (NMDS) of 28S rRNA gene data (A) and of 16S rRNA gene data (B)**. Groupings are based on SIMPROF with complete linkage clustering at 95% confidence at 30% (28S) and 60% (16S) similarities. 2D stress was 0.21 **(A)** and 0.14 **(B)**.

Extraction methods were explicitly compared using the MoBIO 10 g and SARDI 10 g samples. Overall community structure was significantly different between extraction methods (PERMANOVA, monte-carlo, 28S: *P* = 0.002, 16S: *P* = 0.001). Furthermore, all diversity estimates were significantly larger in the 10 g MoBIO extraction for the fungal community, but did not differ significantly for the bacterial community data (**Table 1**). The OTU data showed that only the total fungal OTUs were different between the two methods (**Table 3**). While the replicate dispersion between the two extractions methods for the 10 g samples were similar (**Table 2**) for both the fungal and bacterial communities in the two extractions, they were distinctly separated from each other in NMDS ordinations (**Figure 4**). The extraction method differences illustrated in the fungal and bacterial community ordinations are also apparent in cluster analyses as the SARDI extractions cluster independently from most other samples (Supplementary Figures S2A,B in Supplementary Data). In total, the 10 g SARDI and 10 g MoBIO extractions shared 97.0% of bacterial and 76.1% of fungal sequences contained within 56.4 and 25.1% of the total OTUs, respectively.

### OTU Abundance Contrasts

Comparison of the number of OTUs to the number of sequences clearly indicates that the gain in the number of OTUs decreases with increasing sample size, such that they become almost all singletons at 100g. The mean OTU abundances for each sample size group were plotted pairwise to identify significant differences in relative abundances for specific taxa. For the bacterial community (Supplementary Figures S3A–E in Supplementary data), comparing 16S rRNA gene OTU abundances of the 0.25 g extractions to all others reveals increasing variability as extraction size increases. This occurs particularly with the 16S rRNA gene OTUs containing a greater number of sequences that may be important factors in discriminating samples or informing biological conclusions. For example, the comparison of 0.25 g with 100 g revealed that 75.0% of OTUs containing more than an average of 50 sequences among replicates were significantly different (*t*-test, *P* < 0.05) while only 16.4% of non-singleton or doubleton sequences containing less than 50 sequences were significantly different. The higher abundance OTUs were generally enriched in abundance in the larger sample size extractions with all pairwise comparisons. The taxonomic composition of these OTUs was varied and largely affiliated to *Acidobacteria*, unclassified Archaea and *Rubrobacter*. The 10 g and 100 g SARDI extractions that clustered closely in NMDS ordination exhibited a strong correlation in OTU abundances (*R*^2^ = 0.93, *P* < 0.01). The weakest OTU abundance correlations were observed between the 10 g MoBIO and 10 g SARDI (*R*^2^ = 0.73, *P* < 0.01) and 100 g SARDI (*R*^2^ = 0.61, *P* < 0.01).

For the fungal data, the overall strength of the correlations using the mean 28S gene OTU abundances among the extractions were weaker than that observed with the 16S rRNA gene data (Supplementary Figures S4A–E in Supplementary data). Correlations of the 0.25 g sample OTU abundances were fairly consistent when compared to the 1 g, 5 g, and 10 g MoBio extractions (*R*^2^ = 0.61, 0.64, 0.61, respectively), but decreased largely with the comparisons to the SARDI 10 g (*R*^2^ = 0.33) and 100 g extractions (*R*^2^ = 0.25). Again, the strongest correlation occurred between the two SARDI extractions (*R*^2^ = 0.83, *P* < 0.01) while the weakest were found in all comparisons against the 100 g dataset (*R*^2^ = 0.25 to 0.35). Comparing the 0.25 g with 100 g showed that 75.9% of OTUs containing more than an average of 20 sequences among replicates were significantly different between extraction sizes (*t*-test, *P* < 0.05) and were constrained to a few orders: 67% belonged to the order *Pleosporales*, 14% to *Hypocreales*, 14% to *Sordariales*, and 5% to *Tremellales*, with classification bootstrap values ranging 70–100% at the genus level. In contrast, only 38.5% of non-singleton or doubleton were significantly different (*t*-test, *P* < 0.05).

## Discussion

### Comparison of Sample Sizes

Based on 16S and 28S rRNA gene sequence analysis, sample size significantly influenced the overall bacterial and fungal community structure as measured by richness, evenness, diversity, and the dispersion among replicates. The largest richness, evenness, and diversity values were associated with the 10 g MoBIO extractions indicating that this soil sample extraction size range is optimal for soil community diversity assessments in this soil type. The dispersion metrics MVDISP, PERMDISP and within-group-average PERMANOVA similarities also show that, among the MoBIO extractions, the 10 g samples exhibited the lowest replicate variability. Sub-replicate dispersions were lower than the dispersion observed among all replicates within a particular extraction size, indicating that spatially close samples were more similar than those from another sub-plot within the same sampled area. These values also decreased with increasing sample size, reflecting the importance of larger extraction sizes even at smaller spatial scales, presumably due to small-scale spatial heterogeneity (microsites). Due to this lower dispersion and in the context of an experimental framework where treatment differences in both soil bacterial and fungal community structure are assessed, the 10 g MoBIO extraction should provide a higher probability of detecting differences, though different soil types and/or niches (e.g., rhizosphere soil) may lead to different results.

For the bacterial and fungal communities, the observation that the more abundant OTUs were more likely to exhibit a significant difference among extraction sizes indicates patchiness in the densities of the dominant taxa. These abundant OTUs appear to be located in “hot spots” within the soil; the smaller extraction sizes access the locally abundant but spatially rare. The correlation plots suggest that the larger samples (e.g., 10 g MoBIO) revealed both the locally abundant/spatially rare and the locally rare/spatially abundant bacterial and fungal OTUs. Rarefaction curves support this interpretation by showing a smaller degree of coverage, especially for the fungi. This patchiness or spatial clustering is often associated with fungi (Horton and Bruns, 2001), due to their association with decomposing organic residues and localized spores/resting structures. Indeed, the smaller proportion of fungal shared sequences contained within half the proportion of OTUs, compared to the bacteria, in the 0.25 g – 10 g MoBio comparison further illustrate this fungal spatial patchiness at a small scale. It has been suggested that the detection of low abundance taxa such as the plant pathogenic fungi such as Cochliobolus sativus may be favoured by the larger sample size for extraction (Song et al., 2015). Also, a larger sample size will assist in reducing errors in predicting risk categories for soilborne fungal and nematode diseases based on pathogen inoculum measures from large fields (Ophel-Keller et al., 2008).

We argue that the conclusions on sample size presented by Ranjard et al. (2003) and discussed in a review by Lombard et al. (2011) are not wholly applicable to high throughput sequencing. Specifically, that the use of large soil samples are suitable for the description of the overall soil community structure while large numbers of small samples are more appropriate for a determination of local microbial diversity. Earlier sample size studies were based on less robust techniques such as ARISA (Ranjard et al., 2003) or DGGE (Ellinsøe and Johnsen, 2002; Kang and Mills, 2006) are limited in their ability to assess the ‘rare biosphere’ (Sogin et al., 2006). While dominant members may mask the signatures of minority populations using these techniques, our results suggest that sufficiently deep amplicon sequencing overcomes these limitations by revealing minority populations in the context of the overall community structure. This is supported by the higher diversity indices and larger number of non-singleton/doubleton OTUs in the 10 g sample. Moreover, if dominant populations did indeed mask the more rare OTUs then we would expect more unique OTUs in the 0.25 g sample compared to the 10 g sample. However, we found the opposite through presence/absence analyses; 1191 and 2414 unique fungal OTUs and 1074 and 1657 unique bacterial OTUs in the 0.25 and 10 g samples, respectively. Thus, these data suggest that larger soil samples should not directly bias against identification of new bacterial strains.

### Comparison of Extraction Methods

The comparison of extraction methods using the 10 g MoBio and 10 g SARDI soil extractions showed that while fungal community richness, evenness and diversity was significantly greater in the MoBIO extraction, these differences were not significant in the bacterial community. This is despite the finding that the SARDI extraction recovered significantly more total fungal OTUs. The number of shared sequences between the 10 g MoBIO and 10 g SARDI for both bacteria (97.0%) and fungi (76.1%) suggest that the extraction methods are most comparable for bacterial community analyses.

Indeed, the number of shared sequences between extraction methods was similar to those found between the 0.25 and 10 g MoBio extractions (98.0%-bacteria, 83.8%-fungi), indicating an overall influence of extraction method. The sample dispersion and within group similarities indicate that both methods yielded equally reproducible replicate results. The interaction between the number of OTUs recovered and diversity measures is reflected in the discrepancy of the extraction method that recovered the most fungal OTUs in both the complete data and the non-singleton-doubleton (non-S-D) data. While SARDI recovered the most total fungal OTUs, a larger proportion of these OTUs were rare, leading to a low number of non-S-D OTUs. In this non-S-D data, the number of OTUs recovered from the 1 g, 5 g, and 10 g MoBio extractions were similar.

The taxonomic composition of the bacterial OTUs showed that no specific lineages exhibited large differences in relative abundances (or presence/absence in the more abundant OTUs) among the different size MoBIO and SARDI extractions. In contrast, there were some differences observed among specific fungal lineages. The 0.25 g extractions had higher abundance OTUs containing unclassified *Eukaryota Incertae sedis*, unclassified Glomeromycota and Agaricomycetes. In contrast, both SARDI extractions (10 g and 100 g) resulted in higher abundances of OTUs classified as *Alternaria*, unclassified Pleosporales and *Cercophora*. Members of the Pleosporales group, including *Alternaria* spp. and *Cercophora* spp., produce thick-walled, melanized spores. The recovery of DNA from environmental samples requires efficient cell lysis, especially from spores and other microbial resting structures. The SARDI DNA extraction method was originally standardized/calibrated to extract DNA from resting structures such as nematode cysts and fungal spores from soil samples (Ophel-Keller et al., 2008). The bead-beating intensity in the MoBIO protocol may not be as efficient in lysing these structures, resulting in lower relative abundances. Overall, these differences resulted in the low correlation between the MoBio and SARDI extractions. While the bead-beating SARDI and MoBIO PowerSoil^®^ methods have been previously shown to result in very similar plant root DNA extraction efficiencies, as assessed by quantitative PCR (Haling et al., 2011), the aforementioned differences in OTU abundances associated with each extraction method, especially for the fungal data, would likely lead to differing biological conclusions, especially where explicit taxonomic associations are made.

## Conclusion

In this study we found that soil sample size still plays a role in these rather homogenous soils that were collected during the non-crop season, without the rhizospheric influence of the growing crop, with uniform physical and chemical attributes, management histories, and aboveground crop responses. Nonetheless, these soil cores (and soil subsamples) still represented a range of microhabitats, including the crop residue detritusphere, the rhizospheric legacy from previous crops, the aggregatosphere and other microsites providing unique microbial habitats (Beare et al., 1995). Hence, the larger samples that encompassed a more even distribution of these habitats (microsites) revealed higher microbial diversity with lower replicate variation. However, these results may not apply to other, more specific microbial habitats or other soil types, especially soils with large aggregate structures. For example, the plant rhizosphere microbial community is considered to be more uniform in distribution (Hinsinger et al., 2009). In this circumstance, a smaller soil sample size may be adequate to cover the lower heterogeneity but also necessary to target this smaller habitat. In addition, highly structured soils would likely require soil screening in order to improve homogenization for DNA extraction.

In all, both sample size and extraction method significantly impacted fungal and bacterial community compositions as revealed by high throughput sequencing. This illustrates the essential requirement for transparency and consistency in extraction methods when comparing studies. While the 10 g sample reveals higher diversity, less dispersion among replicates, and more depth of taxa information, the value of the resolution gained needs to be considered relative to (1) the variation of the features/attributes of the system under study, (2) the resolution needed to answer the question and (3) extraction costs.

## Author Contributions

Study design was performed by JT, VG, and RP. Field sampling was carried out by VG. Lab work and data collection was performed by RP and JY. Data interpretation was performed by RP, VG, JT, and JY. All authors contributed to manuscript preparation.

## Conflict of Interest Statement

The authors declare that the research was conducted in the absence of any commercial or financial relationships that could be construed as a potential conflict of interest.

## Footnotes

1 http://www.biology.duke.edu/fungi/mycolab/primers.htm

2 http://icer.msu.edu/hpcc

3 http://rdp.cme.msu.edu

4 http://www.ebi.ac.uk/ena/
