# Supplementary figures and images for: Size Matters: Assessing Optimum Soil Sample Size for Fungal and Bacterial Community Structure Analyses Using High Throughput Sequencing of rRNA Gene Amplicons
Source: Front Microbiol. 2016 Jun 2;7:824. doi: 10.3389/fmicb.2016.00824 (PMC4889595; doi:10.3389/fmicb.2016.00824)

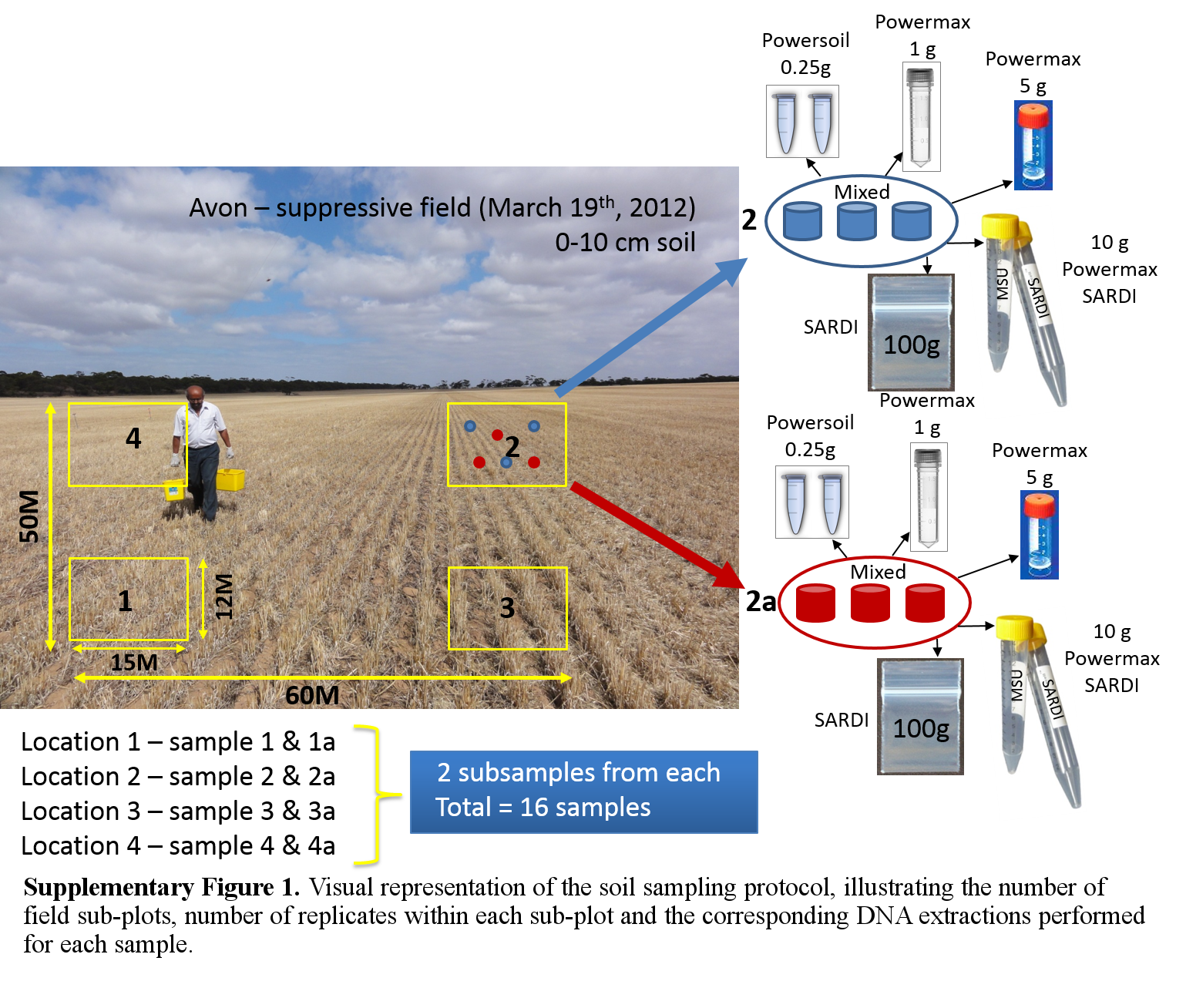

Supplement: Supplementary file 1 [file Image_1.TIF]

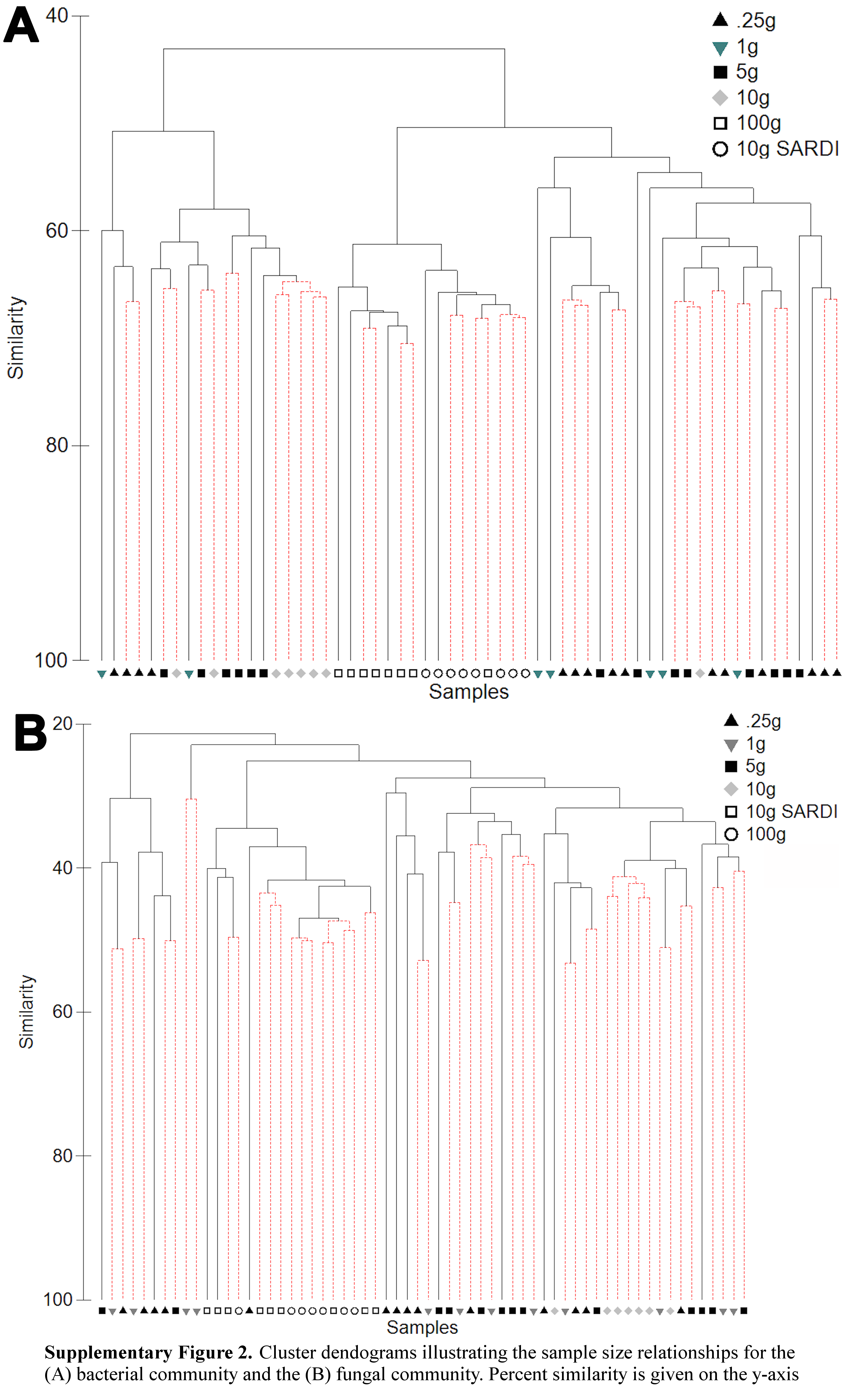

Supplement: Supplementary file 2 [file Image_2.TIF]

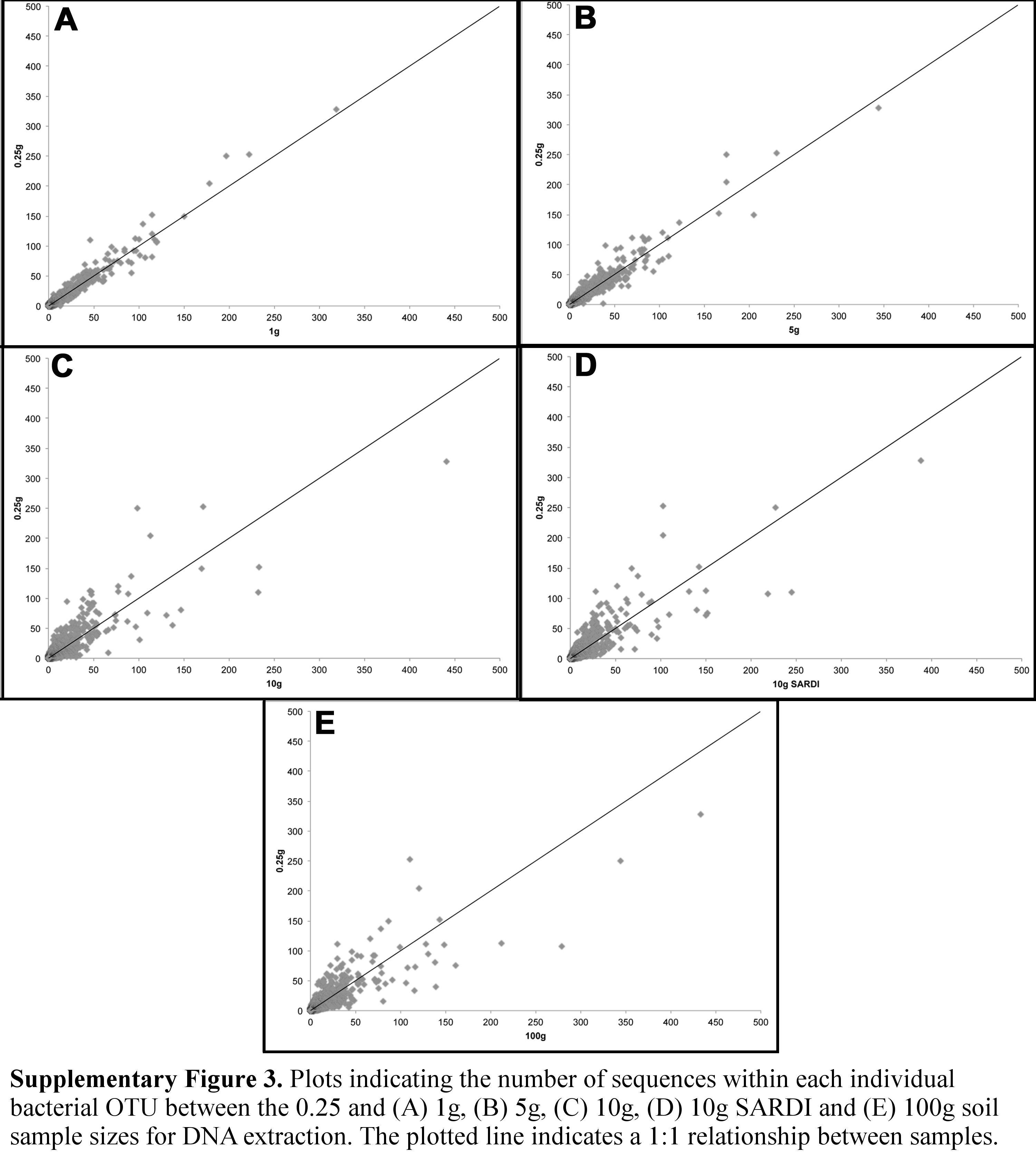

Supplement: Supplementary file 3 [file Image_3.TIF]

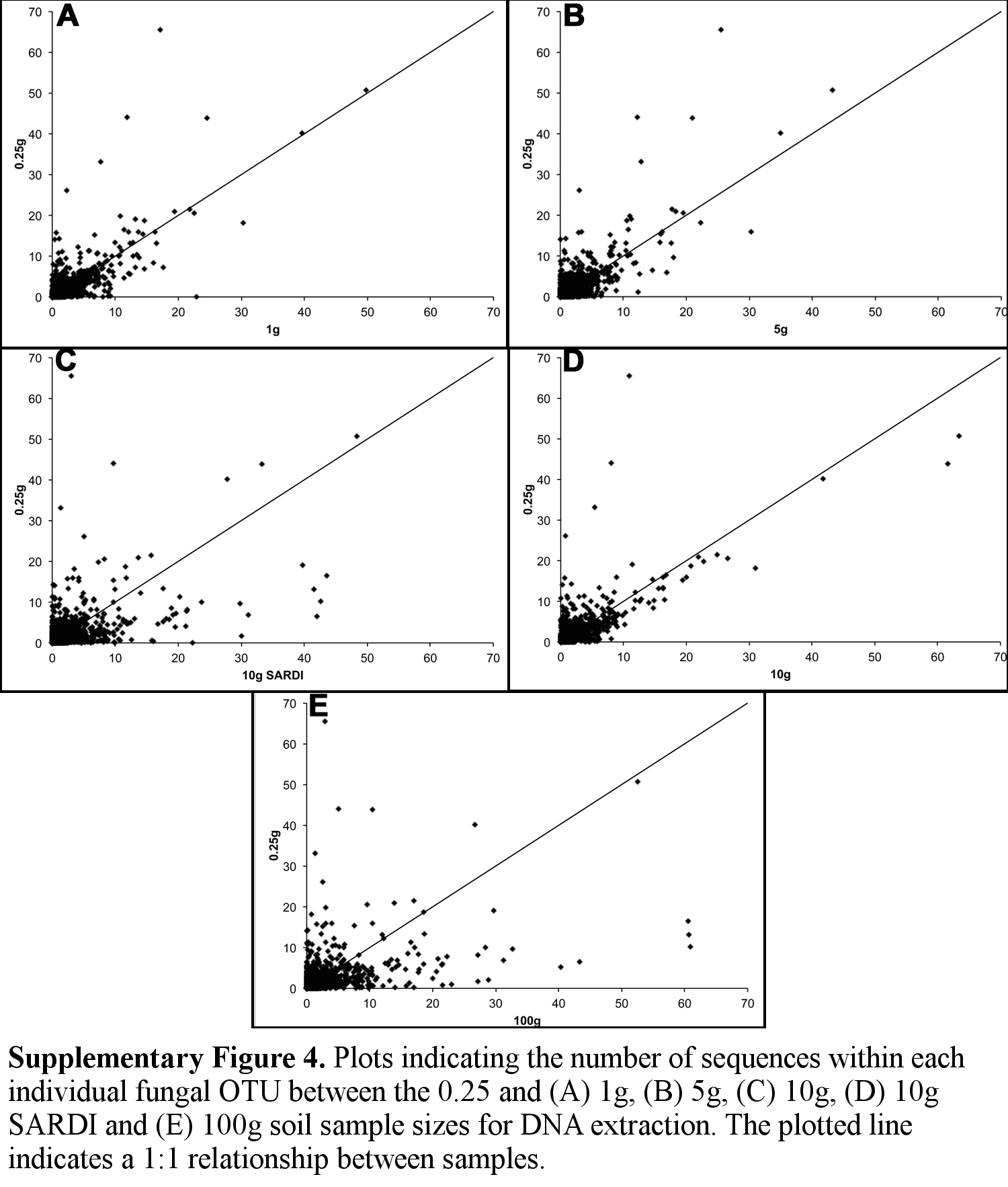

Supplement: Supplementary file 4 [file Image_4.TIF]
